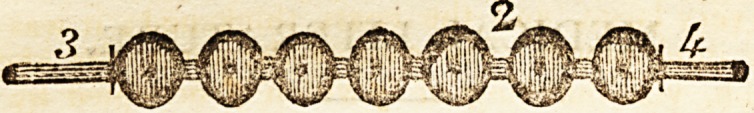# Oriental Instruments for Curing Rheumatism

**Published:** 1817-02

**Authors:** J. Johnson

**Affiliations:** St George's Square, Portsea


### *Oriental Instruments for curing Rheumatism.*

SOME thirty years ago, a gentleman, on his return from the East Indies, (a Mr. Cochrane) called at Haslar Hospital, and presented the physician of that institution a pair of instruments (of which the above cuts convey a representation) which he had seen used in the Eastern world for the cure of chronic rheumatism. The instrument, No. 1, consists of a wooden cylinder, on one end of which is fixed a pretty hard-stuffed ball, covered with leather, and of a moderate size. The second instrument consists also of a cylindrical piece of wood, on which are strung a number of smooth wooden balls, like beads on a wire. The mode of using them for the cure of rheumatism is this: With the ball of the instrument (No. 1) the pained limb or part is beaten, with more or less force, according to its degree of sensibility or tenderness, till the surface exhibits a blush; then the instrument (No. 2) is taken, and being held by the hands of an assistant at No. 3 and 4, the balls are rolled up and down over the said surface, with more or less weight, according to the feelings of the patient. The operation may last a quarter of an hour or more; and is to be repeated every day, till the patient is recovered. The limb is to be bound up pretty tightly with a calico bandage after the process is finished.

This plan of treating chronic rheumatism was actually put in practice under Dr. Lind, of Haslar, and continued for more than a year, when it was gradually dropped; not, however, from its inutility, for in many cases it was evidently beneficial.

From hence, it appears, that Dr. Balfour's mode of curing chronic rheumatism is not new, even in this country; and it is certain that the practice has been pursued time immemorial in the Eastern world. It is probable that Dr. Balfour would find the plan pointed out in this paper, an improvement on his own, and that as a mean of combating an obstinate disease, it is not undeserving the notice of the profession in general.

St George's Square, Portsea, Dec. 1, 1816. J. JOHNSON.

## Figures and Tables

**Figure f1:**
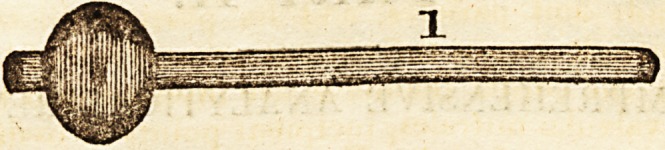


**Figure f2:**